# 

**Published:** 1887-07

**Authors:** 


### INDEX.

### A

PAGE.

Angell, Edward B., M.D. Rochester Pathological Society..... 23
Antiseptic Dressing, Lister's Latest.......................... 30
Association, The New York Physicians' Mutual Aid........... 44
Arsenic in Skin Diseases........ 46
Association, Buffalo Medical and Surgical................ ... 64
Autopsy of King Louis of Bavaria 72
Antipyrin, The Ineligibility of, in Pneumonia.................. 88
Auvard. Relaxation of the Perineum and Vulva during Confinement. Translated by E. B. Angell, M. D..... ... ... 160
Antipyrine, The Proper Use of.. 180
Association, New York State Medical . ...............190, 240
Antifebrin, a New Antipyretic... 230
Advertising Operations in the Daily Papers................. 238
Alexander's Operation, by H Mynter, M.D....... ....... 241
Analysis of Two Hundred and Fifty Autopsies on Drunkards.. 266
Armstrong, Dr E. R., Pregnancy Nephritis........ ... ..... 297
Antifebrin or Acetanilide....... 358
Antiseptics, Comparative Value of 368
Alimentation, Artificial......... 369
Alcoholization of Wines........ 372
Asthma, Tobacco.... ........ 373
Acetanilide or Antifebrin, the Physiological and Therapeutic Action of, by Dr. Weill........ 400
Antifebrin in the Treatment of Phthisis....... .......... 465

### B

PAGE.

Bailey, Wm. C., M. D., Graves' Disease..................... 7
Bronchitis, Chronic ............ 168
Bisulphide of Carbon in Pulmonary Affections, by Dr. Jainn Guerra Estapé. Translated by F. R. Campbell, M.D. ........ 228
Bandage, The Use of an Abdominal, in the Second Stage of Labor.......... ........... 271
Bacteriotherapy................ 315
Bright's Disease, Treatment of.. 361
Bacteria, Study of Atmospheric. 372
Burns, The Treatment of........ 374
Bromides, Peacock's........... 379
Brochin, Dr. A. Is Pulmonary Consumption Contagious?..... 450
Bovine Tuberculosis, its Communicability.................. 513

### C

Clairvoyante Diagnosis ......... 552
Clark, Simeon T., A M., M.D., Report of a Case of Chronic Endartaritis ...... ......... 19
Chrysarobin for Infantile Eczema, Internal Administration of... 31
Chinaman vs. The Sanitarian.... 34
Cæsarean Operation, Sänger's... 36
Carnrick's Soluble Food ..... .. 37
Chorea .......... . ....... .... 75
Childbed, The Necessity of Preparatory Treatment for ....... 79
Caffeine, some of the Therapeutic Effects when Hypodermically Administered ................ 80
Congress. The Ninth International Medical................ 94

PAGE.

Comma Bacillus of Asiatic Cholera, A Reply to, etc. Geo. W. Lewis, Jr., A.M., M.D........ 107
Campbell, F. R., M.D., A New Tæniafuge, Translated by..... 119
Cocaine, The Other Side of—The Bad Side..................... 132
Coryza, Remedies for. ......... 136
Colleges Buffalo. ............. 139
Cervix Uteri, Two Cases of Granular Degeneration of the ..... 177
Croup, Antiseptic Treatment of.. 184
Conklin, Dr. W. L., Mental Influence in Disease .............. 206
Campbell, F. R., M.D. Acute Yellow Atrophy of the Liver, with Report of Case................ 219
Colden's Liquid Beef Tonic..... 240
Communications to the Editor.. 255
Cholera Infantum .......... ... 264
Cocaine in Labor.... .......... 265
Coca Preparations, As to the Efficacy of ..................... 268
Cord, Late Ligation of the, by J. W. Keene A M, M D........ 337
Cervix, The Prevention and Immediate Treatment of Lacerated, by C. C. Frederick, M D. 344
Cocaine, The Effect of Large Doses of, upon the Central Nervous System ........... .......... 366
Clinic, Dr H. Mynter's Surgical, at the General Hospital. ..... 393
Cocaine in the Incoercible Vomitings of Pregnancy........... 423
College, The Annual Commencement of the Buffalo Medical... 425
College, Buffalo Dental......... 431
Consumption, Dr. Bergeon's Method of Treating, by Means of Gaseous Enemata, by Dr. Byron H. Daggett.... ....... 447
Consumption, Another Remedy for .......................... 558
Consumption, Pulmonary, Is it Contagious? by Dr. A. Brochin 450
Coca as a Cardiac Tonic ... ... 466
Campbell, F. R., A.M., M.D. The Causes and Prevention of Infantile Diarrhœal Diseases.... 431
Certificate, A Death............ 502
Carnrick's Soluble Food, Utility of ........... ...... ...... 507
Cathartics, Notes respecting Special. ........................ 511
Cocaine ........................ 512

### D

Disease, A New Parasitic, of Children. Perleche........... ... 68
Diphtheria not a Sewer Gas Disease........ ............... 78
Disease A, Supposed to be New. Perleche..................... 86
Davidson, A. R., M.D., Notes on Diseases of the Skin.......... 111
Diphtheritic Croup, Two Cases of Intubation of the Larynx for. H. D Ingraham, M.D......... 116
Discovery, Highly Important.... 166
Diseases, Diagnosis of Infantile. 174
Digestion, The Influence of Drugs upon Stomach........ ....... 176
Disease, Mental Influence in. Dr. W. L. Conklin ............... 206
Davidson, A. R., M.D., Sycosis Non-Parasitica............... 215
Dysentery, Something Old and New in the Treatment of Acute, by A. B. Fordyce, M.D....... 251
Debierre, Dr. History of Ozone in Therapeutics. Translated by F R. Campbell, M D ....... 259
Drugs, The Action of, in the Movement of the Stomach......... 263
Diphtheria, Treatment of ....... 265
Dyspepsia, Congenital Hereditary Atonic. .. ................... 269
Doses, Memorizing.............. 278
Drugs, Influence of, Given to Nurses or Mothers on their Suckling Infants............. 319
Discoveries, Late............... 320
Druggists, Untrustworthy....... 361
Diabetes, Experimental..... ... 365
Diarrhœa, The Antiseptic Treatment of Summer .... ....... 370

PAGE.
Davidson, A. R., M.D. Religio, Mores, Cultura .. .... ...... 433
Daggett. Dr. Byron H. Dr. Bergeon's Method of Treating Consumption by Means of Gaseous Enemata.................. .. 447
Diseases, The Causes and Prevention of Infantile Diarrhœal, by F. R. Campbell, A.M., M.D... 481

E

Elsner. H. L. M D. Abscess of Gall-Bladder.................. 538
Endartaritis, Report of a Case of Chronic. S. T. Clark. A.M, M.D............ .......... 19
Erysipelas of the Larynx........ 33
Ergot The Proper Use of, in Obstetrical Practice............. . 55
Examinations, Entrance for Medical College.......... .. 92
Eczema of the Scalp ........... 127
Earache.......................... 167
Epilepsy, Theory, Prognosis and Treatment of. ...... ....... 185
Estapé, Dr. Gainn Guerria Bisulphide of Carbon in Pulmonary Affections. Translated by F. R. Campbell, M. D........... 228
Eucalyptol, Hypodermic Injection of, in Phthisis............ 321
Electricity. The Role of, in Tabes 357
Epispadias in the Female........ 308
Euphorbia Drumondii........... 372
Epilepsy, The Pathology and Treatment of.............. ... 407
Education, State Control of Medical.......................... 477
El Kellah...................... 501
Erie County Society, Meeting June 15, 1887... ........... 548

F

Frederick, C. C., M.D. The Watery Discharges of Pregnant Women....................... 154
Fever, Yellow. Its Transmission by the Culex Mosquito.... ... 183

PAGE.
Fordyce, A. B., M. D. Something Old and New in the Treatment of Acute Dysentery..... 251
Fell, Geo. E., M.D Vaginismus 289
Frederick, C. C., M.D. The Prevention and Immediate Treatment of Lacerated Cervix..... 344

G

Gall-Bladder, Abscess of.. .... 538
Graves' Disease. Wm. C. Bailey, M.D......................... 7
Gargle, An Antiseptic.......... 131
Gray, John Purdue ............ 283
Good Samaritan Eye and Ear Infirmary...... ................ 133
Gouley, J W. S., M.D. A Protest against Indiscriminate Meatus Cutting.... ....... .. 349
Gonorrhœa, Latent............. 554

H

Heart, On Nervous Palpitation of the, and its Treatment........ 81
Hamilton, Frank Hastings, M.D., LL.D.................... .... 90
Hernia, Treatment of. Wm. H. Heath, M.D................. 97
Heath, Wm. H., M.D., Treatment of Hernia............... 97
Hematoscopy ................. 124
Hemorrhoids, Forced Dilatation in the Treatment of .......... 167
Heart Trouble, The Diagnosis of Organic ..................... 178
Hubbell, Alvin A., M.D., Congenital Occlusion of the Posterior Auris...... .... .......... 193
Hyoscyamine in Delirium Tremens.......... ......... ... 280
Honor, A Deserving. ..... 285
Hæmoglobinuria, Paroxysmal. Translated by F. R. Campbell, M.D.... .. ............... 312
Heat, Utilization of Earth's Internal....... ............... 352
Health Board in Italy........... 353
Hygiene, A Proposed School of.. 354

PAGE.

Homœopathy, as Regarded by one of its Leaders................ 359
Hygiene of the Hair. By G. Thos. Jackson, M.D.......... 453
Hemorrhage, Alarming, after Tonsilar Incision.............. 463

**I**

Iodide of Potassium in the Treatment of Infantile Broncho Pneumonia.................. 30
Ingraham, H. D., M.D. Two Cases of Intubation of the Larynx for Diphtheric Croup.. 116
Iodol, On Pomade and Solution of....... ...... ...... 126
Iodide of Sodium vs. Iodide of Potassium................. .. 128
Ingraham, H. D., M.D. Intubabation of the Larynx for Croup 226
Insanity, Music in the Treatment of...................... 229
Insanity, Increase of, in the State of New York ...... ....... 316
Iron, The Unrecorded Injury from the Continued Use of Large Doses of ...... ......... 317
Insanity, Classification of....... 385
Irrigation, Intra-Uterine and Vaginal. By Thos. Lothrop, M.D......................... 385
Iodine, Goitres and Interstitial Medication with.............. 423
Ingraham, H. D., M D. Intubation of the Larynx........... 494
Infection, Late Puerperal.... ... 513

Journal, The.................... 40
Jackson, G. Thos, M.D., Hygiene of the Hair .. .............. 483

Kefir.... ....... ......... ..... 264
Keene, J. W., A.M., M.D, Late Ligation of the Cord.......... 337

**L**

Lewis, Jr., Geo. W., A.M., M.D., The Bacillus of Typhoid Fever. 1

PAGE.

Lister's Latest Antiseptic Dressing ......................... 30
Library of Prof. C. C. F. Gay.... 43
Lewis, Jr., Geo. W., A.M., M D., A Reply to, etc................ 107
Liver, Acute Yellow, Atrophy of the with Report of Case. F. R. Campbell, M.D................ 219
Larynx, Intubation of the, for Croup, H. D. Ingraham, M.D. 226
Lesions. Rare, Produced by Brom. of Potassium................. 363
Lactosuria ................... 367
Law School, Buffalo Law Department of Niagara University... 374
Lothrop, Thos., M.D., Intra-Uterine and Vaginal Irrigation .... 385
London Students, The Proposed New Medical Degree for...... 415
Larynx, Intubation of the. By H. D Ingraham, M.D........ 494

**M**

Metranoikter, a New Instrument 546
Massage, the Physiological Effects of.......... ........ 39
Myelitis of the Dorsal Region, Accompanying Pregnancy..... 49
Magnetism of the Blood........ 126
Metric System ... ............ 127
Micrococcus of Soft Chancre... 136
Milk, Unboiled and Boiled...... 140
Medical Profession — Its Nature and Tendencies. Charles G. Stockton, M.D................ 145
Medical Education and Board of Censors....................... 562
McKee, E. S., M.D Treatment of Pruritus Pudendi.......... 158
Menthol as a Local Anæsthetic in Nose and Throat............ 168
Muriate of Pilocarp, The Use and Value of, in Diphtheria ....... 181
Membrana Tympani, the Functions of the, Illustrated by Disease ...................... 182
Miner, Julius T., M.D.......... 233
Mynter's, Dr., Letter........... 240

PAGE.
Menstruation, A New Theory of. 410
Minnesota Medical Practice Act. 460
Malarial Fever, Causation of.. .. 553
Methyl, Chloride of, in Neuralgia 561

N

Negro, The, from a Medical Standpoint. ............... 38
Nitro-Glycerine in Bright's Disease............ ... ....... 270
Neuritis, Peripheral, in Tuberculosis...................... 360
Niagara University, The Annual Commencement of the Medical Department of . ........... 427
Nasal Passages, Some General Rules, which will Determine the Necessity of an Operation in the, by Frank H. Potter M.D. 443
Niagara University, Annual Commencement of................ 466
Neuralgia, Facial, its Operative Treatment ............... ... 510
Niagara University, Law Department of..................... 514

O

Oxygen in Leucæmia and Pseudo-Leucæmia....... .. ....... 184
Ozone, History of, in Therapeutics, by Dr. Debierre. Translated by F. R. Campbell, M.D. 259
Ovulation during Pregnancy.... 263
Overwork and Sanitation in Public Schools................ 276
Obstetrics, Antiseptic Treatment in.... ....... .......... 277
Oil of Turpentine, The Injection of, in Old Sinuses............ 279

P

Pneumonia with Empyemia..... 542
Prescribing Druggists........... 40
Perleche, A New Parasitic Disease of Children ............. 68
Perleche, A Disease Supposed to be New...................... 86
Mynter, H., M.D. Alexander's Operation.................... 240

PAGE.
Meatus-Cutting, A Protest against Indiscriminate, by J. W. S. Gouley, M.D................. 349
Maltine in Phthisis . ......... .... 371
Man, The Chemical............. 408
Potassium Permanganate in Amenorrhœa ...............89–373
Papine, Remarks on the Use of 134
Pneumonia, Digitalis Treatment 137
Pregnant Women, the Watery Discharges of, C. C Frederick, M.D .................. ....... 154
Pruritus Pudendi, Treatment of. E. S. McKee, M.D.... ....... 188
Perineum and Vulva, Relaxation of, during Confinement. Dr. Anward. Translated by E. B. Angell, M.D.......... .. .... 160
Pneumonia, Infections.......... 165
Pruritus Vulvae, An Application for ......................... 167
Posterior Nares, Congenital Occlusion of the. Alva A. Hubbell, M.D... ..............193–335
Potter, Dr. Frank H............ 239
Petroleum Emanations, The Effect of, on Health............ 272
Pasteur and his Methods........ 284
Pregnancy Nephritis. By Dr. E. R. Armstrong................ 297
Pasteurism in Russia........... 314
Phthisis, The Latest Treatment of 362
Prescriptions, Incompatible..... 413
Parotid Gland, Secondary Inflammation of the........... .... 422
Polypi, Nasal.................. 424
Potter, F. H., M. D. Some General Rules which will Determine the Necessity of, in Operation in the Nasal Passages.... 443
Pyo-Salpinx in its Relation to Puerperal Fever............... 504
Penis, Horny Growth on........ 559

Q

Quinine, To Disguise the Taste of 135

PAGE.

### R

Rochester Pathological Society. Edward B. Angell, M. D...... 23
Rheumatism, Salicylate of Lithia in the Treatment of. ........ 184
Rheumatism, For Chronic...... 265
Rheumatism, Treatment of Acute 267
Reducing Substance in Urine, Resembling Glucose............ 273
Re-organization of the Health Department................... 280
Religio, Mores, Cultura, Doctorate address, by A. R. Davidson, M. D........................ 433
Rochester, Thos. F., M.D., LL.D. 517
Ring, Dr. Wm., Death of....... 523

### S

Sänger's Cæsarean Operation.... 36
Soluble Food, Carnrick's.... 37
Skin, Notes on Diseases of the. A. R. Davidson, M.D. ....... 111
Salol.......................... 133
Saccharine....................... 137
Stockton, Charles G., M.D. The Medical Profession—Its Nature and Tendencies.............. 145
Scarlet Fever and Diphtheria, A Specific for.................. 179
Sycosis Non-Parasitica. A. R. Davidson, M.D.............. 215
Sunstroke..................... 279
Society, Erie County Medical.... 303
Syphilis, Cerebral.............. 322
Saccharine, the Pharmaceutical Uses of.................... 334
Substances, The Various, resembling Membranes found in the Throats of Children.......... 365
Spermatorrhœa in Gonorrhœa... 366
Substitution.................... 379
Skin-Grafting, Medico-Legal Aspects on..................... 408
Sepsis, Virulent, Purulent....... 511
Syphilis and Marriage........... 555

### T

Typhoid Fever, the Bacillus of, by Geo. W. Lewis, Jr., A.M., M.D......................... 1
Tait's, Mr. Lawson, Mistake..... 35
Tumors, Treatment of Malignant, by the Injection of Oil of Turpentine...................... 38
Tæniafuge, A New. Translated by F. R. Campbell, M.D...... 119
Tooth-Powder, An Agreeable Antiseptic...................... 128
Tonsillitis, Treatment of Acute. 166
Test, A New, of Medical College Standing..................... 168
Tooth-Ache, A Cure for, from Dental Caries................ 168
Tuberculosis, The Etiology of... 231
Tendon, Translation of, from Animal to Man.................. 509
Trachea and Lung, Percutaneous Injection of Fluids into...... 560

### U

Urethritis, Treatment of Chronic 39
Uterus, Retroversion of the..... 170
Uric Acid Deposits, The Clinical Significance of. Translated by F. R. Campbell, M.D......... 310
Ulceration, Tubercular, of the Tongue........................ 465
Urethra, Stricture of the Female 560

### V

Vesical Irritation, An Anodyne Use in......................... 37
Vaginismus, by George E. Fell, M.D.......................... 289
Vomiting, The Treatment of Certain Forms of................ 369

### W

Warts.......................... 168
Whooping Cough, the Treatment of............................ 278
Weill, Dr. The Physiological and Therapeutic Action of Acetanilide or Antifebrin............. 400
Whooping Cough, the Immediate Cure of....................... 409
Wound on the Foot Successful ful Transplantation of Frog-Skin upon a Granulating .... 421
Wedding Trip.................... 464

### Z

Zinc Ointment for Varicose Ulcers....................... 128

